# Subchronic safety evaluation of hot-water extract from thinned immature mangos (*Mangifera indica* ‘Irwin’): 90-days oral toxicity study in rats

**DOI:** 10.1016/j.toxrep.2021.05.005

**Published:** 2021-05-12

**Authors:** Hayato Tajiri, Wataru Tanaka, Masakatsu Takashima, Hiroki Matsuyama, Takuya Sugita, Kenta Hidaka, Hiroyuki Sakakibara

**Affiliations:** a Graduate School of Agriculture, University of Miyazaki, Miyazaki, Japan; b Agricultural Administration Section, Saito City, Miyazaki, Japan; c Star-Fruits Company, Ltd., Miyazaki, Japan

**Keywords:** CPK, creatine phosphokinase, MCH, mean corpuscular hemoglobin, MCHC, mean corpuscular hemoglobin concentration, MCV, mean corpuscular volume, NOAEL, no-observed-adverse-effect level, RBC, red blood cell, S.D., standard deviation, TIMEx, hot-water extract of thinned immature mango fruits, No-observed-adverse-effect level (NOAEL), Oral toxicity, Rats, Subchronic toxicity, Thinned immature mango

## Abstract

Thinned immature fruit of the mango tree (*Mangifera indica* ‘Irwin’) are handled as waste. In this study, we conducted a 90-days toxicity study in male and female Sprague Dawley rats to evaluate the safety of a hot-water extract of thinned immature mango fruits (TIMEx) administered by oral gavage at doses of 500, 1000 and 2500 mg/kg body weight/day. Treatment did not result in death or changes in the behavior or external appearance of the animals. No alterations were observed in hematological or serum chemical parameters, urinalysis, food consumption, body weight gain or organ weights at the end of the treatment period, with the exception of higher mean corpuscular volume in male rats that received high doses and lower serum creatine phosphokinase levels in female rats that received medium doses. Under the conditions of this study and based on the toxicological endpoints evaluated, the no-observed-adverse-effect level (NOAEL) for TIMEx was 2500 mg/kg/day. The findings indicate that TIMEx is safe for consumption and should be investigated as a candidate food.

## Highlights

• Thinned immature mango fruits are usually handled as waste.
• Information is limited about the safety of its daily consumption.
• A 90-days toxicity study of its hot-water extract (TIMEx) was conducted in rats.
• TIMEx is safe for daily consumption and its NOAEL is 2500 mg/kg/day.
• Thinned immature mango fruits will be candidate for functional food.

## Graphical abstract

Thinned Irwin mango fruits were collected at the immature stage and lyophilized. Hot-water extract from thinned immature mango (TIMEx) had no toxic effect on SD rats given daily oral doses of 500, 1000 or 2500 mg/kg for 90 days. No significant alterations in hematological and serum chemical parameters, urinalysis, food consumption, body weight gain, or absolute and relative organ weights were noted in any group. In both sexes, a daily dose up to 2500 mg/kg body weight indicates a NOAEL.

**Figure:**
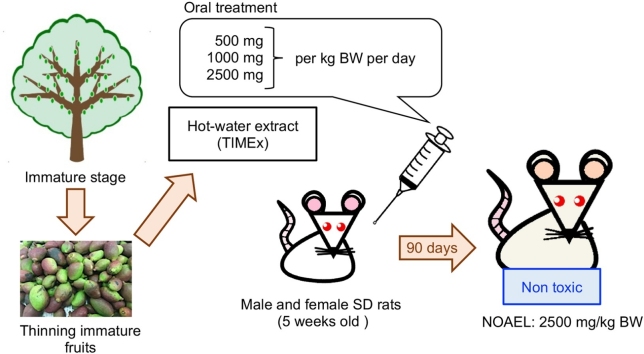

## 1 Introduction

Mango (*Mangifera indica*) is one of the most favored tropical fruits in the world. Global mango production reached about 40 million tons in 2018, an increase of 2.8 percent over 2017 [1]. In addition to dense sweetness, the fruit pulp has high nutritional value for vitamins, dietary fiber and diverse polyphenols [[2], [3], [4]]. Therefore, the mango is referred to as the “King of fruits” [5].

**Fig. 1:**
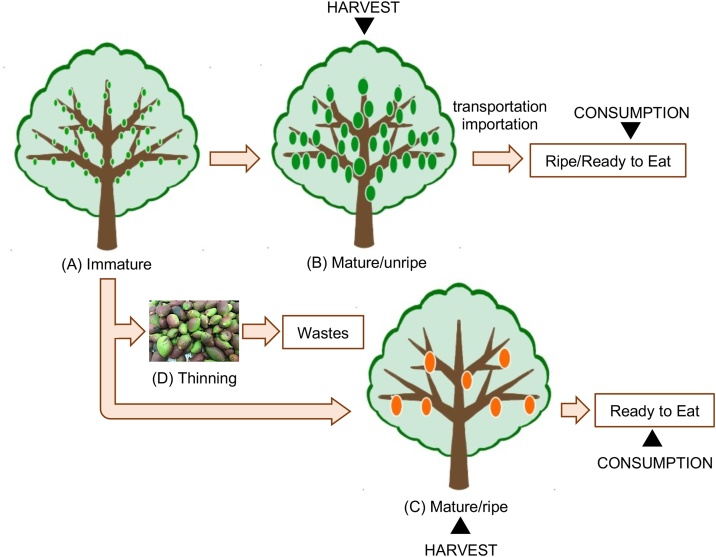
Flowchart of the production of fruits from mango cultivars.

For many fruits, including mango, size is a major factor that determines their yield, marketability and price. An increasing number of fruit per tree correlates with decreases in mean fruit weight and in the proportion of fruit in the larger size grades [6,7]. The mango fruit develops on the tree starting with an immature stage (Fig. 1A). The average weight of this stage is <10 g and the color is green. The fruits then grow and reach the mature size, but the color is still green (mature/unripe stage; Fig. 1B). This stage is the typical harvest point for mangos that are to be exported. During transportation and distribution and finally with the consumer, unripe mangos progress to a ripe/ready to eat stage. Mature/unripe mangos produce ethylene, a naturally occurring ripening hormone and ripen normally on their own [8]. When most of the fruits are thinned during the immature stage, the size and quality of the remaining mango fruits are increased (mature/unripe after thinning stage; Fig. 1C) [9]. Fruit that is harvested when immature will soften, but it will not develop a pleasing flavor indicating that the ripening process will not salvage immature mango fruit [8]. Therefore, most thinned immature mango fruits are handled as waste.

We have recently focused on thinned immature fruits (Fig. 1D) of the Irwin mango cultivar (*Mangifera indica* ‘Irwin’) as an unused natural resource. Important biochemical, physiological and structural changes that affect mainly nutritional and phytochemical composition and produce softening, aroma and flavor modification, and antioxidant capacity, occur during the development stage from unripe to ripe. For example, lipid content increases during ripening, particularly omega-3 and omega-6 fatty acids [3]. Additionally, the Brix value, which represents the dry substance content of squeeze solutions containing mainly sucrose and fructose [10], increases, and acidity, peel strength and pulp firmness decrease during ripening [11], while magnesium levels increase but phosphorus, potassium, calcium and sodium levels decrease with ripening [12]. However, there is insufficient information concerning the safety and eating characteristics of thinned immature mango fruits to enable their use as a natural resource. In this study, we therefore carried out subchronic safety evaluation of hot-water extract from thinned immature mango fruits, which can be consumed whole, body including the peel, flesh, and seed, using a 90-day safety study in rats.

## 2 Materials and methods

### 2.1 Experimental materials and preparations

#### 2.1.1 Immature mango fruits

Immature Irwin mango fruits grown in Miyazaki, Japan, were collected by hand between the middle of February and the end of March 2019, and taxonomically identified on the basis of morphological characteristics by Mr. Kenta Hidaka (Star-Fruits Company, Ltd., Miyazaki, Japan). The weight and size of individual fruits were <25 g and < Φ3 cm (Fig. 1D). The fresh samples were immediately transported to the laboratory, and washed with tap water to remove dirt and dust. The cleaned whole fruits including peel, flesh and seed were rapidly frozen in liquid nitrogen, and then lyophilized using a freeze dryer (FDU-2110, Tokyo Rikakikai Co., Tokyo, Japan). Thinned immature mango powder was obtained using a Knife mill grindomix GM 200 (Verder Scientific Co., Tokyo, Japan), and stored away from light at 4 °C until extraction. All other reagents were of the highest grade available.

#### 2.1.2 Hot-water extraction

Hot-water extraction was conducted according to the modified method for making the hot-water extract from green tea leaf [13]. Briefly, thinned immature mango powder was mixed with ten volumes of water at 95 °C. After agitation for 10 min, filtrate was obtained via filtration through a 5 μm mesh (ADVANTEC No. 2, Toyo Roshi Kaisha, Tokyo, Japan), and then lyophilized using an FDU-2110 freeze dryer. The mango powder yield was 25–28 %. The lyophilized powder of hot-water extract from thinned immature mango (TIMEx) was stored away from light at 4 °C until used in animal experiments.

#### 2.1.3 Preparation of test solutions

Before starting the experiment, we checked the solubility of TIMEx in deionized water, and found that the maximum solubility was about 250 mg/mL. Therefore, this concentration was employed as maximum concentration, and further dissolved at concentrations of 1000 and 500 mg/10 mL in deionized water according to the OECD 408 guideline “Repeated Dose 90-day Oral Toxicity Study in Rodents” [14] and our previous study [15]. After thorough mixing, solutions were administered to rats at 10 mL/kg body weight/day.

### 2.2 Animal experiments

#### 2.2.1 Institutional approval of the study protocols

All animal procedures were approved by the Institutional Animal Care and Use Committee of the University of Miyazaki, Japan (No. 2019-015-01). This study was conducted in accordance with the Japanese Law for the Humane Treatment and Management of Animals (Law No. 105, 1973), which defined animal experimentation as the use of animals for scientific purposes with the consideration of the 3Rs.

#### 2.2.2 Animals and treatments

The study protocol followed modified methods of our previous study [15] according to the OECD 408 guideline “Repeated Dose 90-day Oral Toxicity Study in Rodents” [14]. Briefly, thirty-two 4-week-old Sprague-Dawley rats of each sex (thirty-two males and thirty-two female) were obtained from Japan SLC (Shizuoka, Japan). In this study, groups of eight rats of each sex were employed as similar to the previous reports [16,17]. The animals were housed singly in polycarbonate cages (W270 mm × L440 mm × H187 mm) with paper bedding (Alpha-dri Certified, EPS Ekishin Co., Tokyo, Japan) at 23 ± 2 °C, with 55 ± 10 % humidity and a 12 h light/dark cycle (light period: 9:00 am to 9:00 pm) and with free access to laboratory chow (MF; Oriental Yeast Company, Tokyo, Japan) and deionized water. After 1 week of acclimatization, the animals were randomly divided into four groups. Three groups were orally administered the TIMEx solution once daily during the middle of the light period. Each group received daily 2500 mg/10 mL/kg (high-dose), 1000 mg/10 mL/kg (medium-dose) or 500 mg/10 mL/kg (low-dose). The fourth group received 10 mL/kg of the vehicle (deionized water).

#### 2.2.3 Clinical and physiological observations

All animals were observed twice daily for mortality, general condition, and clinical signs. Any abnormal findings were recorded with respect to symptom, extent, severity, and date of detection. Body weights were measured daily, immediately prior to administration, and food consumption was measured at least three times per week. Water consumption was measured during days 85–88. The effect on locomotor activity was also evaluated according to our previous report [18]. Briefly, on days 75–80, rats were placed in an open-field space (60 cm × 90 cm), and their locomotion and rearing frequency was observed in a 5-min period.

#### 2.2.4 Urinalysis

Each rat was housed individually in a metabolic cage (KN-647, Natsume Seisakusho Co., Tokyo, Japan) during days 78–87, and urine was collected over a period of 24 h. Urine volume was calculated using weight and density, which was analyzed by a urine specific gravity refractometer (MASTER-SUR/JM, Atago Co., Tokyo, Japan). The color and turbidity were evaluated visually. Urinary glucose, total protein, and creatinine levels were analyzed using a Dri-Chem 4000v chemistry analyzer (Fujifilm Co., Tokyo, Japan). Urinary pH was measured with a pH meter (LAQUAtwin pH-11B, HORIBA, Kyoto, Japan).

#### 2.2.5 Hematology and blood chemistry

After administration of TIMEx for 90 days, the rats were fasted for 12 h, and blood samples were taken from the abdominal vein under anesthesia with isoflurane (2.5 %). A 2 mL aliquot was added to a K_2_-EDTA Venoject tube (VP-DK052K05, Terumo Medical Corp., Tokyo, Japan) and allowed to stand at room temperature for 30 min. Hematological parameters were then analyzed using Celltacα MEK-6500 (Nihon Kohden Co., Tokyo, Japan). Next, another aliquot of blood (6 mL) was added to a Venoject tube containing a procoagulant (VP-AL076 K, Terumo Medical Corp.). After standing for 30 min at room temperature, the serum fraction was obtained by centrifugation (1200×*g*, 10 min, room temperature) and stored at −80 °C until analysis. Serum biochemical parameters listed in Table 4 were analyzed using a Dri-Chem 4000v chemistry analyzer.

#### 2.2.6 Necropsy and organ weights

After blood collection, the following organs and tissues were evaluated macroscopically and any abnormalities were recorded: Adrenal gland, duodenum, epididymis, eyes, heart, ileum, jejunum, kidneys, liver, lungs, ovaries, prostate, pancreas, skeletal muscle, skin, spleen, stomach, urinary bladder, uterus, testes, thymus, and thyroid. The following organs and tissues were weighed: Adrenal gland, brain, carcass, heart, kidney, liver, lung, spleen, thymus, thyroid, mesenteric visceral fat, testis, seminal vesicle, ovary and uterus.

#### 2.2.7 Determination of serum cytokine levels

Serum cytokine levels were analyzed using a multiplex biometric enzyme-linked immunosorbent assay according to the manufacturer’s instructions (Rat Cytokine/Chemokine 9-Panel, RECYTMAG-65 K, Millipore, Billerica, MA, USA), for the simultaneous detection and quantitation of interleukin (IL) 4, IL-5, IL-6, IL-10, IL-12, IL-13, IL-18, interferon (IFN) γ and tumor necrosis factor (TNF) α. Their amounts were determined using a multi-analyte profile with MAGPIX (Millipore).

### 2.3 Data analysis

All data are presented as the mean ± standard deviation (S.D.). Statistical analyses were conducted using StatView for Windows (version 5.0, SAS Institute, Cary, NC, USA). One-way analysis of variance (ANOVA) analysis was used for groups stratified by sex. If significant, the Tukey Kramer test for multiple comparisons was applied to compare the control and treatment groups. Results were considered significant if the probability of error was <5%.

## 3 Results

### 3.1 Mortality and clinical signs

TIMEx was orally administered daily for 90 days. The treatment appeared to be well-tolerated. No rat died during the exposure period, and no clinical signs such as diarrhea, hair loss or aberrant activity (locomotion and rearing frequency) were observed (data not shown).

### 3.2 Body weight and food consumption

Body weight gain and food consumption did not differ among the treatment and control groups or between sexes throughout the study period (Table 1, Table 2). In addition, no remarkable differences were observed in water consumption among control, low-, medium- and high-dose groups: 31.5 ± 9.5, 31.6 ± 5.1, 33.7 ± 5.2, and 32.1 ± 4.4 g/day/rat, respectively, for male rats; 25.1 ± 3.6, 24.7 ± 3.1, 28.7 ± 5.4, and 27.4 ± 7.1 g/day/rat, respectively, for female rats.

**Table 1:** Mean body weights of rats administered hot-water extract from unripe mango fruits for 90 days.

days	Males (n = 8)				Females (n = 8)			
	Control	TIMEx (mg/kg body weight/day)			Control	TIMEx (mg/kg body weight/day)		
		500	1000	2500		500	1000	2500
0	182.5 ± 11.4	183.0 ± 15.4	182.7 ± 9.4	183.8 ± 10.6	123.8 ± 10.6	123.5 ± 8.1	123.3 ± 7.3	123.5 ± 6.6
10	219.9 ± 12.9	222.0 ± 20.4	223.0 ± 12.3	222.4 ± 15.4	143.3 ± 12.6	147.2 ± 10.7	144.2 ± 7.2	145.9 ± 10.1
20	292.2 ± 20.2	298.7 ± 24.7	299.0 ± 18.5	299.1 ± 18.2	178.9 ± 16.1	179.9 ± 14.4	177.5 ± 10.1	180.6 ± 17.0
30	355.4 ± 30.3	360.9 ± 28.5	355.5 ± 28.2	357.6 ± 22.6	207.5 ± 18.4	208.6 ± 13.5	207.0 ± 15.1	208.8 ± 21.3
40	398.6 ± 38.3	409.6 ± 31.1	402.2 ± 36.0	402.0 ± 26.9	227.4 ± 19.6	228.4 ± 14.2	228.7 ± 19.0	230.6 ± 25.6
50	429.0 ± 44.1	441.0 ± 30.6	428. ± 38.6	430.4 ± 29.9	243.0 ± 21.1	242.9 ± 16.7	243.4 ± 20.8	245.1 ± 29.4
60	456.5 ± 50.3	465.9 ± 27.6	452.8 ± 42.4	451.1 ± 31.7	253.9 ± 22.3	253.2 ± 19.0	253.2 ± 22.6	256.4 ± 32.2
70	483.4 ± 53.9	492.2 ± 29.7	476.6 ± 45.6	478.9 ± 32.0	261.7 ± 22.2	258.3 ± 20.6	259.9 ± 23.2	262.4 ± 35.3
80	503.0 ± 55.5	513.5 ± 32.5	497.1 ± 22.6	499.9 ± 31.4	268.6 ± 21.2	267.8 ± 20.1	270.6 ± 22.8	271.3 ± 36.4
90	518.5 ± 53.5	530.9 ± 32.5	517.2 ± 47.1	517.6 ± 34.8	276.1 ± 22.4	274.1 ± 20.0	278.3 ± 22.8	277.5 ± 34.6

TIMEx hot-water extract from thinning immature mango. All values represent the mean (in grams) ± S.D. (n = 8). No significant differences were found between control and treated rats (P < 0.05, Tukey Kramer test).

**Table 2:** Mean food consumption by rats administered hot-water extract from unripe mango fruits for 90 days.

days	Males (n = 8)				Females (n = 8)			
	Control	TIMEx (mg/kg body weight/day)			Control	TIMEx (mg/kg body weight/day)		
		500	1000	2500		500	1000	2500
10	15.3 ± 1.7	15.6 ± 1.3	15.2 ± 1.2	15.4 ± 1.2	13.5 ± 1.4	13.8 ± 1.6	13.3 ± 0.8	12.8 ± 1.4
20	16.2 ± 1.4	16.4 ± 1.7	16.2 ± 1.8	16.4 ± 1.0	15.6 ± 1.2	16.3 ± 1.8	15.6 ± 1.6	15.9 ± 2.3
30	15.6 ± 1.5	16.1 ± 1.4	16.2 ± 1.7	16.3 ± 1.5	15.0 ± 1.3	15.4 ± 1.4	15.7 ± 1.8	15.5 ± 2.4
40	16.6 ± 1.9	16.9 ± 1.3	16.3 ± 2.3	16.1 ± 1.2	15.4 ± 1.0	16.3 ± 1.5	16.3 ± 1.8	15.9 ± 2.3
50	15.8 ± 1.8	15.5 ± 1.0	15.2 ± 1.8	15.0 ± 1.2	14.8 ± 1.2	15.4 ± 1.7	14.9 ± 1.6	15.0 ± 2.2
60	16.1 ± 1.8	15.8 ± 1.0	15.6 ± 1.7	15.4 ± 1.1	14.5 ± 0.8	14.8 ± 1.8	14.2 ± 1.4	14.8 ± 2.0
70	16.0 ± 1.5	15.8 ± 1.7	15.7 ± 1.9	15.9 ± 0.8	14.3 ± 1.2	14.5 ± 2.0	14.2 ± 1.5	14.1 ± 2.6
80	15.5 ± 1.4	15.5 ± 1.2	15.5 ± 0.6	15.4 ± 1.1	14.4 ± 0.9	14.9 ± 1.4	14.8 ± 0.8	14.6 ± 2.1
90	14.6 ± 1.0	14.8 ± 1.3	15.1 ± 1.3	14.9 ± 0.8	13.8 ± 1.5	14.1 ± 1.4	14.1 ± 1.4	13.7 ± 1.9

TIMEx hot-water extract from thinning immature mango. All values represent the mean (in grams/rat/day) ± S.D. (n = 8). No significant differences were found between control and treated rats (P < 0.05, Tukey Kramer test).

### 3.3 Hematology and blood chemistry

Mean corpuscular volume (MCV) significantly increased in male rats in the high-dose group (Table 3). This change was not observed in female rats in any group. Assessment of serum biochemistry revealed significant changes in creatine phosphokinase activity in female rats in the medium-dose group (Table 4). This change was not observed in male rats in any group. Other hematology and blood chemistry parameters were not altered in either sex in any group at the end of the 90-day administrated period.

**Table 3:** Hematological parameters of rats administered hot-water extract from unripe mango fruits for 90 days.

	Males				Females			
	Control (n = 8)	TIMEx (mg/kg body weight/day)			Control (n = 8)	TIMEx (mg/kg body weight/day)		
		500 (n = 8)	1000 (n = 8)	2500 (n = 7)^1^		500 (n = 8)	1000 (n = 8)	2500 (n = 8)
WBC (10^3^/μL)	6.51 ± 0.80	8.13 ± 0.45	7.28 ± 0.65	7.76 ± 0.20	5.18 ± 1.03	5.53 ± 1.63	4.78 ± 0.75	5.88 ± 1.34
RBC (10^6^/μL)	8.25 ± 0.25	8.32 ± 0.12	8.33 ± 0.05	8.16 ± 0.13	7.73 ± 0.40	7.45 ± 0.17	7.44 ± 0.56	7.50 ± 0.29
HGB (g/dL)	13.9 ± 0.3	13.9 ± 0.2	14.1 ± 0.1	14.1 ± 0.3	14.7 ± 0.7	14.4 ± 0.4	14.2 ± 1.1	14.3 ± 0.2
HCT (%)	38.7 ± 1.2	39.0 ± 0.6	39.8 ± 0.3	39.5 ± 0.7	40.8 ± 2.1	39.5 ± 0.8	39.2 ± 2.9	39.8 ± 1.1
MCV (fL)	46.9 ± 0.3	46.8 ± 0.3	47.8 ± 0.4	48.4 ± 0.2*	52.9 ± 0.8	53.0 ± 1.4	52.7 ± 0.8	53.1 ± 1.6
MCH (pg)	16.9 ± 0.2	16.7 ± 0.2	16.9 ± 0.2	17.3 ± 0.1	19.1 ± 0.4	19.3 ± 0.5	19.1 ± 0.3	19.1 ± 0.6
MCHC (g/dL)	36.0 ± 0.4	35.7 ± 0.2	35.4 ± 0.2	35.8 ± 0.2	36.1 ± 0.8	36.4 ± 0.4	36.2 ± 0.5	35.9 ± 0.7
PLT (10^4^/μL)	84.4 ± 12.0	94.4 ± 4.1	97.1 ± 2.8	90.8 ± 2.3	94.0 ± 9.3	95.1 ± 9.4	90.5 ± 7.6	96.1 ± 5.6
RDW-CV (%)	12.0 ± 0.1	12.0 ± 0.1	12.2 ± 0.2	12.0 ± 0.3	12.5 ± 0.4	12.3 ± 0.8	12.3 ± 0.5	12.4 ± 0.6
RDW-SD (fL)	22.5 ± 0.2	22.5 ± 0.2	23.3 ± 0.3	23.3 ± 0.5	26.5 ± 1.1	26.2 ± 2.1	26.0 ± 1.3	26.2 ± 1.9
PCT (%)	0.53 ± 0.08	0.59 ± 0.03	0.62 ± 0.02	0.56 ± 0.01	0.56 ± 0.05	0.57 ± 0.04	0.54 ± 0.03	0.57 ± 0.04
MPV (fL)	6.36 ± 0.07	6.20 ± 0.06	6.31 ± 0.07	6.19 ± 0.05	6.01 ± 0.26	6.04 ± 0.24	5.99 ± 0.23	5.88 ± 0.19
PDW (%)	15.5 ± 0.4	14.8 ± 0.2	15.1 ± 0.1	15.2 ± 0.1	15.4 ± 0.3	15.4 ± 0.4	15.5 ± 0.3	15.4 ± 0.2

TIMEx hot-water extract from thinning immature mango. All values represent the mean ± S.D. HCT, hematocrit; HGB, hemoglobin; MCH, mean corpuscular hemoglobin; MCHC, mean corpuscular hemoglobin concentration; MCV, mean corpuscular volume; MPV, mean platelet volume; PCT, plateletcrit; PDW, platelet distribution wide; PLT, platelet; RBC, red blood cell; RDWCV, red cell distribution-CV; RDWSD, red cell distribution-SD; WBC, white blood cell. ^1^One data was missing, because one blood sample was coagulated before analysis. *Significantly difference vs Control group (P < 0.05, Tukey Kramer test).

**Table 4:** Serum biochemistry parameters of rats administered hot-water extract from unripe mango fruits for 90 days.

	Males				Females			
	Control (n = 8)	TIMEx (mg/kg body weight/day)			Control (n = 8)	TIMEx (mg/kg body weight/day)		
		500 (n = 8)	1000 (n = 8)	2500 (n = 7)^1^		500 (n = 8)	1000 (n = 8)	2500 (n = 8)
ALT (U/L)	32.3 ± 3.6	36.9 ± 4.8	38.3 ± 10.5	32.0 ± 4.3	57.3 ± 16.0	54.2 ± 14.9	59.9 ± 28.0	50.6 ± 21.6
AST (U/L)	51.4 ± 10.9	63.6 ± 10.1	72.5 ± 30.7	49.6 ± 3.5	91.7 ± 21.3	86.5 ± 26.5	107.1 ± 74.1	91.1 ± 36.0
Alkaline phosphatase (U/L)	511 ± 95	605 ± 228	549 ± 165	531 ± 153	403 ± 149	462 ± 151	399 ± 151	457 ± 133
Amylase (kU/L)	3.2 ± 0.6	3.4 ± 0.7	3.5 ± 0.8	2.7 ± 0.5	2.0 ± 0.6	2.0 ± 0.6	1.8 ± 0.4	2.3 ± 0.5
Leucine aminopeptidase (U/L)	56 ± 3	53 ± 3	55 ± 4	54 ± 5	64 ± 23	55 ± 5	55 ± 5	56 ± 9
LDH (U/L)	134 ± 16	118 ± 21	141 ± 37	164 ± 32	199 ± 57	171 ± 26	186 ± 72	162 ± 22
Creatine phosphokinase (U/L)	88 ± 10	92 ± 16	110 ± 41	89 ± 10	118 ± 33	92 ± 18	88 ± 14*	103 ± 10
Cholinesterase (U/L)	0.48 ± 0.07	0.47 ± 0.05	0.50 ± 0.08	0.53 ± 0.05	0.94 ± 0.14	0.91 ± 0.15	0.93 ± 0.14	0.85 ± 0.11
Total bilirubin (mg/dL)	0.13 ± 0.07	0.10 ± 0.04	0.11 ± 0.04	0.16 ± 0.07	0.17 ± 0.05	0.16 ± 0.09	0.18 ± 0.08	0.12 ± 0.03
Uric acid (mg/dL)	0.79 ± 0.16	0.67 ± 0.06	0.90 ± 0.43	0.63 ± 0.07	0.65 ± 0.10	0.61 ± 0.06	0.60 ± 0.12	0.64 ± 0.11
Glucose (mg/dL)	195 ± 18	198 ± 13	213 ± 31	201 ± 16	174 ± 17	179 ± 21	185 ± 27	172 ± 24
Blood urea nitrogen (mg/dL)	19.5 ± 1.4	18.8 ± 2.9	18.0 ± 1.6	20.1 ± 2.3	21.4 ± 5.4	21.3 ± 2.4	18.6 ± 3.4	17.1 ± 2.6
Creatinine (mg/dL)	0.34 ± 0.17	0.42 ± 0.26	0.36 ± 0.19	0.33 ± 0.22	0.25 ± 0.03	0.32 ± 0.26	0.21 ± 0.02	0.21 ± 0.05
Total cholesterol (mg/dL)	66 ± 13	58 ± 7	65 ± 14	71 ± 20	101 ± 12	103 ± 21	103 ± 16	84 ± 18
HDL cholesterol (mg/dL)	47.0 ± 9.9	40.3 ± 5.9	46.9 ± 10.5	52.0 ± 13.6	64.0 ± 8.4	67.5 ± 9.9	67.9 ± 7.0	57.1 ± 11.8
Non-HDL cholesterol (mg/dL)	18.6 ± 4.9	17.2 ± 2.1	17.6 ± 3.9	18.6 ± 6.2	37.1 ± 8.8	35.7 ± 12.0	34.8 ± 10.5	27.3 ± 7.2
Triglycerides (mg/dL)	137 ± 57	123 ± 24	133 ± 49	114 ± 40	52 ± 15	60 ± 29	66 ± 27	46 ± 18
Albumin (g/dL)	3.5 ± 0.2	3.4 ± 0.1	3.5 ± 0.3	3.3 ± 0.3	3.8 ± 0.3	3.9 ± 0.6	3.9 ± 0.3	3.6 ± 0.2
Total protein (g/dL)	6.4 ± 0.2	6.4 ± 0.2	6.4 ± 0.3	6.2 ± 0.5	6.6 ± 0.3	6.7 ± 0.5	6.7 ± 0.5	6.4 ± 0.3
NH_3_ (μg/dL)	97 ± 26	100 ± 45	147 ± 112	94 ± 64	165 ± 126	145 ± 97	210 ± 80	182 ± 76
Phosphorus (mg/dL)	6.0 ± 0.6	6.0 ± 0.5	6.5 ± 1.0	6.3 ± 0.3	5.8 ± 0.7	6.0 ± 0.4	5.6 ± 0.8	5.8 ± 0.5
Calcium (mg/dL)	10.5 ± 0.5	10.5 ± 0.4	10.7 ± 0.2	10.6 ± 0.4	10.4 ± 0.3	10.6 ± 0.4	10.6 ± 0.4	10.3 ± 0.3
Magnesium (mg/dL)	2.0 ± 0.2	1.9 ± 0.2	2.0 ± 0.1	1.9 ± 0.1	2.1 ± 0.2	2.1 ± 0.2	2.0 ± 0.2	2.0 ± 0.1
Sodium (mEq/L)	132 ± 5	136 ± 7	133 ± 5	133 ± 4	133 ± 14	136 ± 5	138 ± 3	133 ± 8
Potassium (mEq/L)	3.7 ± 0.4	3.7 ± 0.2	3.7 ± 0.2	3.6 ± 0.2	3.4 ± 0.5	3.5 ± 0.3	3.6 ± 0.1	3.5 ± 0.3
Chloride (mEq/L)	94 ± 4	96 ± 4	93 ± 3	93 ± 2	93 ± 12	96 ± 4	95 ± 3	94 ± 6

TIMEx hot-water extract from thinning immature mango. All values represent the mean ± S.D. ALT, alanine aminotransferase; AST, aspartate aminotransferase; LDH, lactate dehydrogenase. ^1^One datum was missing, because one blood sample was coagulated before analysis.

* Significantly difference vs Control group (P < 0.05, Tukey Kramer test).

### 3.4 Urinalysis

No significant variations in pH, total protein or glucose levels were found by urinalysis in any treatment group (Table 5).

**Table 5:** Urinalysis findings of rats administered hot-water extract from unripe mango fruits for 90 days.

	Males				Females			
	Control	TIMEx (mg/kg body weight/day)			Control	TIMEx (mg/kg body weight/day)		
		500	1000	2500		500	1000	2500
pH	7.8 ± 0.4	7.7 ± 0.2	7.8 ± 0.4	7.7 ± 0.4	7.7 ± 0.4	7.3 ± 0.4	7.5 ± 0.3	7.6 ± 0.5
Total protein (g/g creatinine)	2.27 ± 0.89	2.18 ± 0.30	2.46 ± 0.49	2.10 ± 0.26	2.62 ± 1.13	1.70 ± 0.73	2.13 ± 0.60	2.34 ± 1.17
Glucose (g/g creatinine)	0.11 ± 0.06	0.11 ± 0.02	0.12 ± 0.04	0.11 ± 0.04	0.14 ± 0.04	0.12 ± 0.04	0.13 ± 0.04	0.12 ± 0.04

TIMEx hot-water extract from thinning immature mango. All values represent the mean ± S.D. (n = 8). No significant differences were found between control and treated rats (P < 0.05, Tukey Kramer test).

### 3.5 Necropsy

No visible alterations were associated with TIMEx treatment, with the exception of sporadic findings, including self-injury to the tail in one control male rat. Such injury was not observed in any other groups, including female rats (data not shown).

### 3.6 Organ weights

There were no significant differences in absolute and relative organ weights among sexes and treatment groups, although visceral fat weights in both male and female rats tended to decrease as the TIMEx dose increased (Table 6).

**Table 6:** Absolute and relative organ weights of rats administered hot-water extract from unripe mango fruits for 90 days.

	Males				Females			
	Control	TIMEx (mg/kg body weight/day)			Control	TIMEx (mg/kg body weight/day)		
		500	1000	2500		500	1000	2500
Absolute organ weights (g)								
Body weight	525 ± 51	534 ± 32	525 ± 52	525 ± 36	276 ± 22	275 ± 20	278 ± 23	278 ± 35
Carcass	399 ± 40	421 ± 25	401 ± 33	401 ± 27	209 ± 17	208 ± 14	206 ± 14	209 ± 24
Liver	14.4 ± 2.7	14.6 ± 1.4	14.1 ± 1.7	14.3 ± 1.6	6.99 ± 0.51	7.20 ± 0.49	7.15 ± 0.73	7.24 ± 0.89
Kidney	2.74 ± 0.22	2.94 ± 0.24	2.81 ± 0.18	2.94 ± 0.24	1.61 ± 0.14	1.71 ± 0.16	1.64 ± 0.06	1.72 ± 0.19
Adrenal	0.057 ± 0.007	0.057 ± 0.008	0.062 ± 0.006	0.055 ± 0.006	0.066 ± 0.011	0.068 ± 0.002	0.067 ± 0.015	0.069 ± 0.017
Spleen	0.848 ± 0.129	0.830 ± 0.081	0.772 ± 0.106	0.850 ± 0.135	0.522 ± 0.043	0.582 ± 0.075	0.556 ± 0.049	0.557 ± 0.099
Heart	1.26 ± 0.14	1.31 ± 0.08	1.31 ± 0.11	1.26 ± 0.07	0.788 ± 0.044	0.863 ± 0.064	0.825 ± 0.036	0.832 ± 0.088
Lung	1.58 ± 0.17	1.55 ± 0.12	1.63 ± 0.12	1.63 ± 0.22	1.01 ± 0.07	1.11 ± 0.08	1.07 ± 0.05	1.11 ± 0.10
Visceral fat	25.5 ± 6.2	24.6 ± 5.0	23.5 ± 8.9	22.1 ± 3.7	13.0 ± 3.9	13.3 ± 4.3	12.1 ± 4.0	11.4 ± 4.4
Thymus	0.550 ± 0.082	0.516 ± 0.103	0.535 ± 0.120	0.558 ± 0.081	0.373 ± 0.081	0.318 ± 0.023	0.331 ± 0.049	0.332 ± 0.093
Thyroid	0.018 ± 0.006	0.019 ± 0.002	0.020 ± 0.003	0.020 ± 0.004	0.016 ± 0.002	0.015 ± 0.002	0.013 ± 0.001	0.016 ± 0.002
Brain	2.01 ± 0.08	2.01 ± 0.05	1.91 ± 0.16	2.00 ± 0.11	1.89 ± 0.09	1.83 ± 0.15	1.90 ± 0.09	1.90 ± 0.14
Testis	2.18 ± 0.49	2.03 ± 0.53	2.27 ± 0.21	2.18 ± 0.49	–	–	–	–
Seminal vesicle	5.72 ± 0.55	6.25 ± 0.58	6.06 ± 0.30	6.23 ± 0.41	–	–	–	–
Ovary	–	–	–	–	0.140 ± 0.026	0.161 ± 0.031	0.134 ± 0.018	0.154 ± 0.16
Uterus	–	–	–	–	0.490 ± 0.118	0.617 ± 0.163	0.659 ± 0.179	0.551 ± 0.073

Relative organ weight (g/100 g body weight)								
Carcass	76.1 ± 0.9	76.9 ± 1.0	76.4 ± 1.6	76.4 ± 1.1	75.6 ± 2.1	75.4 ± 1.4	74.3 ± 2.7	75.5 ± 1.3
Liver	2.74 ± 0.28	2.73 ± 0.12	2.69 ± 0.13	2.73 ± 0.15	2.53 ± 0.06	2.62 ± 0.16	2.58 ± 0.26	2.61 ± 0.11
Kidney	0.523 ± 0.029	0.550 ± 0.030	0.539 ± 0.051	0.560 ± 0.022	0.586 ± 0.050	0.621 ± 0.035	0.593 ± 0.056	0.622 ± 0.040
Adrenal	0.011 ± 0.001	0.011 ± 0.001	0.012 ± 0.001	0.011 ± 0.002	0.024 ± 0.004	0.025 ± 0.002	0.024 ± 0.005	0.058 ± 0.004
Spleen	0.161 ± 0.013	0.156 ± 0.012	0.147 ± 0.011	0.161 ± 0.021	0.190 ± 0.015	0.211 ± 0.016	0.201 ± 0.018	0.200 ± 0.019
Heart	0.240 ± 0.011	0.244 ± 0.021	0.252 ± 0.030	0.240 ± 0.014	0.286 ± 0.013	0.314 ± 0.012	0.298 ± 0.022	0.301 ± 0.013
Lung	0.301 ± 0.021	0.293 ± 0.021	0.311 ± 0.025	0.310 ± 0.041	0.379 ± 0.033	0.402 ± 0.015	0.387 ± 0.028	0.401 ± 0.025
Visceral fat	4.82 ± 0.86	4.63 ± 0.83	4.38 ± 1.23	4.19 ± 0.54	4.67 ± 1.25	4.78 ± 1.51	4.30 ± 1.33	4.04 ± 1.11
Thymus	0.105 ± 0.015	0.095 ± 0.022	0.104 ± 0.031	0.106 ± 0.014	0.135 ± 0.031	0.117 ± 0.016	0.119 ± 0.013	0.119 ± 0.026
Thyroid	0.0034 ± 0.0010	0.0035 ± 0.0004	0.0038 ± 0.0007	0.0038 ± 0.0009	0.0057 ± 0.0006	0.0053 ± 0.0006	0.0048 ± 0.0004	0.0058 ± 0.0006
Brain	0.385 ± 0.036	0.377 ± 0.024	0.366 ± 0.046	0.383 ± 0.034	0.687 ± 0.036	0.667 ± 0.050	0.686 ± 0.064	0.692 ± 0.079
Testis	0.416 ± 0.089	0.378 ± 0.096	0.435 ± 0.059	0.413 ± 0.076	–	–	–	–
Seminal vesicle	1.09 ± 0.08	1.14 ± 0.11	1.16 ± 0.07	1.19 ± 0.09	–	–	–	–
Ovary	–	–	–	–	0.051 ± 0.007	0.059 ± 0.009	0.048 ± 0.006	0.056 ± 0.005
Uterus	–	–	–	–	0.178 ± 0.045	0.224 ± 0.056	0.237 ± 0.062	0.202 ± 0.045

TIMEx hot-water extract from thinning immature mango. All values represent the mean ± S.D. (n = 8). No significant differences were found between control and treated rats (P < 0.05, Tukey Kramer test).

### 3.7 Serum cytokine levels

**Fig. 2:**
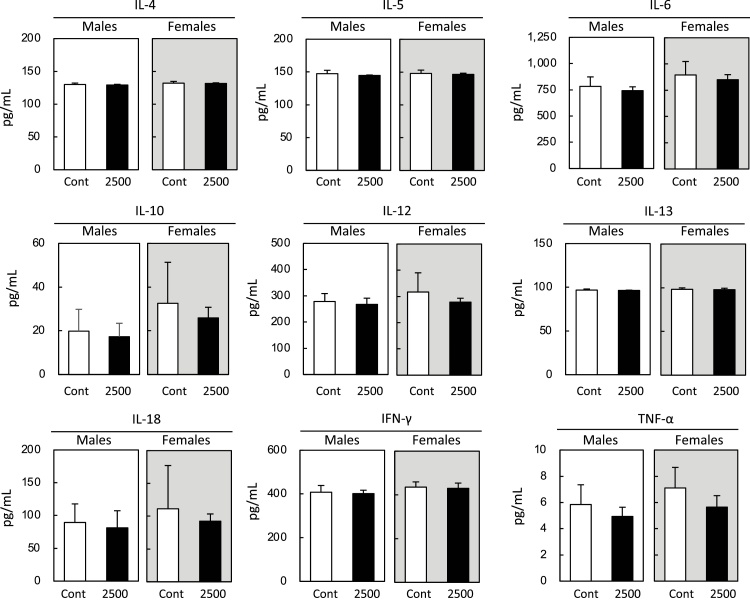
Serum cytokine levels in rats administered TIMEx for 90 days. Hot-water extract from thinning immature mango (TIMEx) at a doge of 2500 mg/kg body weight/day was orally administered to male (white background) and female (gray background) for 90 days. Control group (Cont) was same volume of vehicle (deionized water) was administered. Individual interleukin (IL), interferon (IFN) γ, tumor necrosis factor (TNF) α levels were analyzed. Data indicate as mean ± S.D. (n = 8). There was no significantly deference between Cont and 2500 mg TIMEx/kg body weight groups among every parameter (P < 0.05, Tukey Kramer test).

Fig. 2 shows the serum levels of various cytokines in male and female rats following a 90-day TIMEx treatment at a dose of 2500 mg/kg body weight/day. Notable alterations were not observed in both sexes.

## 4 Discussion

The size and quality of mango fruits are increased if a large number of fruits is thinned during the immature stage [9]. However, thinned immature mangos do not undergo ripening [8], making them unsuitable for consumption as raw fruits. Therefore, they are usually handled as waste. However, we have considered the possibility of processed food materials from thinned immature mangos, for example hot-water extraction to produce a tea. We have focused on the practical values of this unused natural resource; however, safety information for the resource was insufficient. Therefore, we conducted the present study to evaluate the effects of consuming TIMEx. Firstly, the acute oral toxicity test was investigated according to the OECD 423 guideline [19]. Administration of a single dose of 2500 mg TIMEx/kg body weight to male Sprague-Dawley rats did not affect body weight gain or food consumption and did not cause diarrhea or loss of hair during 14 days of observation (data not shown). Subsequently, the repeated dose 90-day oral toxicity study was carried out. There were no deaths or changes in behavior or external appearance among the rats dosed daily with TIMEx at 500 mg/kg body weight/day (low dose), 1000 mg/kg body weight/day (medium dose) and 2500 mg/kg body weight/day (high dose) for 90 days. No significant alterations in hematological or serum chemical parameters, urinalysis, food consumption, body weight gain, or absolute and relative organ weights were noted in any of the dose groups, with a few exceptions.

Higher MCV was noted in male rats in the high-dose group. MCV is a measure of the average volume or size of a red blood cell, and changes with average red blood cell (RBC) size [20]. Higher MCV is observed with elevations in mean corpuscular hemoglobin (MCH) and mean corpuscular hemoglobin concentration (MCHC). For example, intraperitoneal administration of gold nanoparticles to rats increased these three corpuscular parameters [21]. Chronic alcohol consumption increases MCV and MCHC compared with control patients [22]. In our results, neither MCH nor MCHC were changed by the administration of any TIMEx concentration. On the other hand, similar results obtained in this study, in which were higher MCV, equilibrium MCH and MCHC, were reported in anemic rats induced by phenylhydrazine [23]. However, in our study, hemoglobin levels were not reduced compared with the control group. In addition, blood levels of anemia-related inflammatory cytokines, including IFN-γ, TNF-α, IL-6 and IL-10 [24,25], were not affected by the daily administration of 2500 mg TIMEx/kg body weight in both sexes. Therefore, we concluded these effects were sporadic, but not toxic.

The serum biochemistry showed lower creatine phosphokinase (CPK) activity in female rats in the medium-dose group. When muscle damage occurs, muscle cells release CPK into the blood; therefore, CPK is an accurate indicator of muscle damage [26] and abnormal values of CPK occur in a variety of extracardiac disorders [27]. This suggests that lower CPK activity observed in this study might be because of immobility. However, the locomotor activity was not different between the vehicle and medium-dose groups in female rats. Additionally, AST and ALT activities, which are typical biomarkers for hepatic injury, change concomitantly with CPK after muscle injury [28]. However, we did not observe any changes in serum AST or ALT activities in any group. Furthermore, the CPK findings in female rats in the medium-dose group did not indicate a dose-response relationship, and hence were sporadic but not toxic.

## 5 Conclusions

A 90-day TIMEx treatment was well-tolerated by Sprague Dawley rats. No significant changes in clinical signs, hematology, blood chemistry, or urinalysis were observed. Many 90-day repeated dose studies using rodents produce a no-observed-adverse-effect level (NOAEL) [29]. Our results indicated that a daily dose of TIMEx up to 2500 mg kg body weight may therefore be a NOAEL. The animal dose should not be extrapolated to a human equivalent dose by a simple conversion based on body weight. Instead, a body surface area normalization method has been proposed to convert an animal dose to the equivalent human dose, which is often represented in mg/m2; the dose should be multiplied by the *K_m_* factor of 37 for adult humans and 6 for rats [30]. When using this conversion factor, the daily 2500 mg TIMEx/kg body weight in rats is multiplied by the *K_m_* factor of 6 for rats and then divided by the *K_m_* factor of 37 for adult humans. This calculation results in an adult human equivalent dose for TIMEx of 405 mg/kg, which equates to a daily 24,324 mg dose of TIMEx for a 60 kg person. Recently, many functional aspects of mature mango have been reported; for example, antioxidant properties, and anti-diabetic and anti-obese effects [[31], [32], [33], [34]]. Therefore, we consider TIMEx to be a novel beneficial food material candidate. The next logical step is to design studies to evaluate if TIMEx administration improves certain health conditions or prevents the onset of adverse health conditions, including obesity, diabetes and metabolic disorders.

## Transparency document

The Transparency document associated with this article can be found in the online version.

## Declaration of Competing Interest

The authors report no declarations of interest.
